# Investigation of Mold Flux Entrapment in Deep Oscillation Mark of Interstitial-Free Steel Shell Using Mold Simulator

**DOI:** 10.3390/ma17061435

**Published:** 2024-03-21

**Authors:** Xiong Yan, Wanlin Wang, Lejun Zhou, Xiaocan Zhong, Hongliang Lin, Xiaokang Liu, Sibao Zeng, Liwu Zhang

**Affiliations:** 1 School of Metallurgy and Environment, Central South University, Changsha 410083, China; yanx89@foxmail.com (X.Y.);; 2 National Center for International Research of Clean Metallurgy, Central South University, Changsha 410083, China; 3 Guangdong Guangqing Metal Technology Co., Ltd., Yangjiang 529500, China; 4 Shandong Shiheng Special Steel Group Co., Ltd., Feicheng 271612, China

**Keywords:** slag entrapment, mold flux, oscillation mark, mold simulator, IF steel

## Abstract

The slag entrapment defect has become a big issue for the IF steel casting process. In this study, the mechanism of mold flux entrapment in deep oscillation mark of an IF steel shell was studied by a high-temperature mold simulator. Results show that both temperature and heat flux in a copper mold become lower when mold flux B with lower melting and viscosity is used, compared with these when mold flux A with higher melting and viscosity is used. The average thickness of the slag film for mold fluxes A and B is 1.31 mm and 1.63 mm, and the consumption of them is 0.33 kg/m^2^ and 0.35 kg/m^2^, respectively. The shell for mold flux A exhibits sharper oscillation marks, while the shell for mold flux B has shallower oscillation marks. These deeper oscillation marks capture the mold flux by overflow of molten steel at the meniscus, which finally produces the slag entrapment defect in the shell.

## 1. Introduction

Interstitial-free (IF) steel, with a high plastic strain ratio, low yield strength, good formability and high strain rate sensitivity, is widely used for car body panels, such as hoods or doors [1,2]. Except mechanical properties, a high surface quality that is defect-free is also a necessary requirement for IF steel [3,4]. In the process of continuous casting, the mold flux floating on the top of molten steel can be entrapped into the shell and leads to a slag entrapment in the slab [5,6,7]. These entrapped slags cannot be eliminated but transform into elongated silver defects on surface after rolling. The silver defects originated from the slag entrapment have become one of the biggest issues, which causes the degradation of IF panels.

The mold flux entrapment behavior has been investigated by many researchers. Cho et al. [8] studied the changes in transient flow and surface slag behavior in molds with different nozzle port angles using a 3D large eddy simulation (LES) numerical model, and results showed that the undesirable flow variations, due to the improper nozzle port angle, could cause severe instability at the interface of liquid flux/molten steel, which resulted in slag entrapment. Mallikarjuna et al. [9] investigated the impact of immersion depth of a submersed nozzle (SEN) on the mold flow profile and slag entrapment through a 0.5 scaled water model. They suggested that surface velocity and slag entrapment decreased with the increase in SEN depth. Zhang et al. [10] observed the entrapped inclusions beneath the surface of ultra-low carbon steel slab by ASPEX microscope, and they found that those inclusions were mainly located at the oscillation marks on slab. Sengupta et al. [11] also found similar results. Due to the very low content of carbon, the solidification temperature of IF steel is quite high [12], which causes an easily forming solidified hook at the meniscus in the mold. The solidified long hook is also an inducement of deep oscillation mark and entrapment of mold flux [5,13,14]. In addition, a small quantity of Al and Ti is also contained in IF steel. These active metals can be oxidated and transformed into oxide inclusions, which is another defect source of slivers on rolling products [15,16].

Although mold flux entrapment has been investigated by using numerical models, water models, microscopes, etc., high-temperature simulation experiments were rarely conducted. In addition, previous works were mostly focused on the entrapment behaviors on the interface of liquid flux/molten steel based on fluid flow, with very few of them involved the systematic study of the intrinsic interactions between slag infiltration, slag film in the gap between the mold wall and shell, heat transfer, formation of an oscillation mark on the shell and slag entrapment at the oscillation mark. Therefore, in this study, the mechanism of mold flux entrapment in the deep oscillation mark of an IF steel shell was investigated using a mold simulator. Hopefully, the results obtained here can provide a guide for reducing the silver defects and improving the surface quality of IF panels.

## 2. Experimental Materials and Apparatus

### 2.1. Materials

The chemical composition of the IF steel, which complies with the GB/T 5213-2008 standard [17], is provided in Table 1. Two mold fluxes were used in this experiment. Among them, mold flux A is a typical commercial mold powder for casting IF steel. Mold flux B is a newly designed mold powder with a lower melting temperature, higher break temperature and lower viscosity through adding more fluxes (Na_2_O and F) and increasing the basicity and MgO content. The reason why the mold flux with a lower melting temperature, higher break temperature and lower viscosity was designed is because that this kind of mold flux is a benefit for the formation of a liquid mold flux layer on top of molten steel and the infiltration of mold flux into the gap between the mold wall and initial solidified shell, consequently forming a thicker and uniform slag film in the gap. In addition, a mold flux with a higher breaker temperature also means the formation of a thicker crystalline layer in the slag film. Overall, a thicker and uniform slag film with a thicker crystal layer can prevent the formation of severe oscillation marks and reduce the entrapment of slag, due to its higher thermal resistance. Their composition and high-temperature properties are shown in Table 2.

### 2.2. Mold Simulator

**Figure 1:**
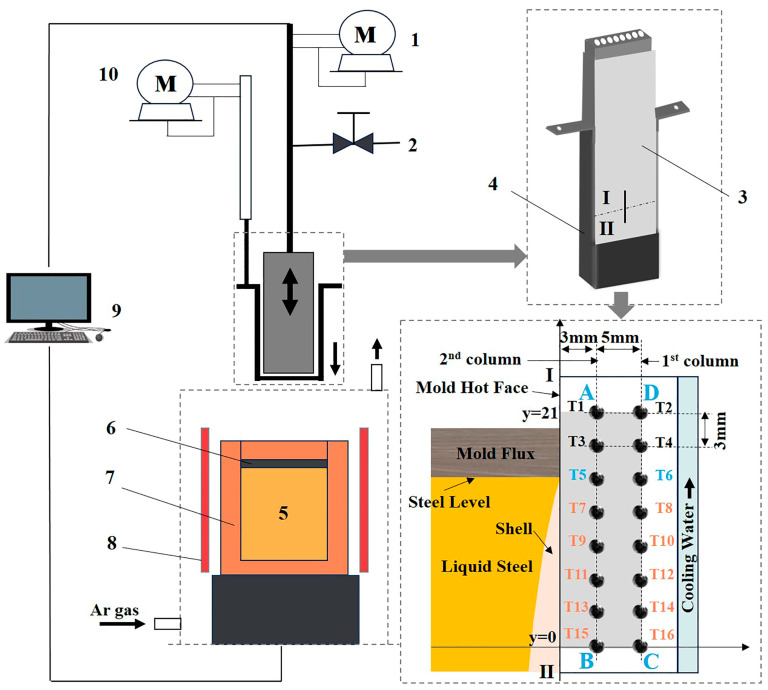
Schematic of the high-temperature mold simulator: 1 the drive of oscillation, 2 the inlet of cooling water, 3 copper mold, 4 extractor, 5 molten steel bath, 6 mold flux, 7 furnace body, 8 induction coil, 9 control and acquisition system, 10 extractor drive.

The entrapment of mold flux in the deep oscillation mark of an IF steel shell was studied using a high-temperature mold simulator. The schematic figure of this device is exhibited in Figure 1. It consisted of a mold oscillator system (1), water cooling system (2), copper mold (3,4), heating furnace (5,6,7,8), control/acquisition system (9) and shell withdrawal (extractor) device (10). It has been successfully used to study the initial solidification of molten steel in casting mold; the details of the high-temperature mold simulator system have been presented in a previous paper [18].

During the experiment, 25 kg of IF steel, which has been cut into smaller blocks with size of 10 mm × 10 mm × 10 mm, was loaded into the furnace. Later, 0.6 kg mold powders were also filled in the furnace after the steel was melted completely. The melting process was protected by inletting pure Ar gas. After that, the copper mold together with extractor, which works as a cover, was descended into the molten bath. Both the copper mold and extractor were held for seconds to make sure that the initial solidified shell on the surface of the mold formed before extraction. Then, the extractor drew the initial solidified shell at a speed of 1.2 m/min downward to simulate the casting process. A slag film also formed in the same process, due to the infiltration of liquid flux from the molten bath top into the solidified shell and copper mold gap. When the casting process ended, the copper mold together with extractor were lifted up from the molten bath, and then, they were cooled in air.

In the whole process, the copper mold oscillated sinusoidally as the real mold in a continuous caster. Its oscillation frequency and stroke are 164 cpm and 4.6 mm, respectively, according to Equations (1) and (2). The parameters of the simulation experiment are consistent with the industrial casting process, and their specific values are listed in Table 3.

(1) $$S=a_{1}+a_{2}V_{c}$$

(2) $$f=a_{3}+a_{4}V_{c}$$

where S is the stroke, mm; *f* is the mold frequency, cpm; $a_{1}$, $a_{2}$, $a_{3}$, $a_{4}$ are the constants of the mold, which are 3 mm, 1.34 × 10^−3^ min, 140 cpm, 20 m^−1^, respectively; and $V_{c}$ is the casting speed, m/min.

**Figure 2:**
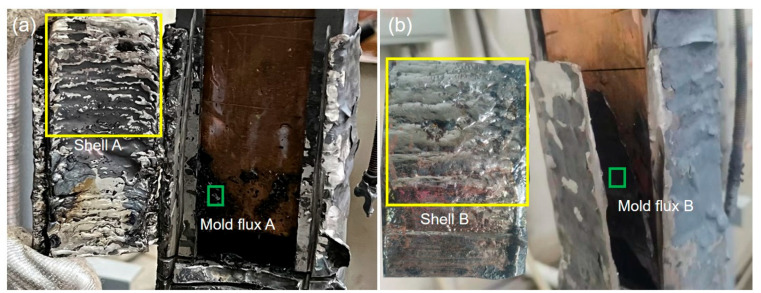
Solidified shell and slag film after the simulation experiment: (**a**) mold flux A; (**b**) mold flux B.

After the simulation experiment, the shell and slag film were cut down from the extractor (Figure 2) for further analyses. The morphology and composition of the two shells and two slag films were characterized using a light microscope (OM, Leica DM4 M, Wetzlar, Germany), scanning electron microscope (SEM, JSM-6360LV, Tokyo, Japan) and X-ray Energy dispersion spectrometer (EDS, EDX-GENESIS 60S, San Diego, CA, USA). In addition, the heat flux across from the molten steel to the mold wall was calculated using a model of two-dimensional inverse heat conduction problem (2D-IHCP), based on the measured temperature by the embed thermocouples, as in a previous paper [18].

## 3. Results and Discussion

### 3.1. Temperature in Mold

**Figure 3:**
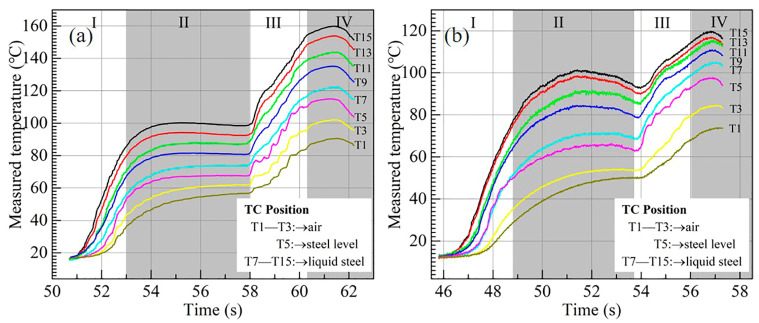
The measured temperature: (**a**) mold flux A; (**b**) mold flux B.

There are two row-embed thermocouples in the copper mold as shown in Figure 1, and Figure 3 shows the temperature in the mold 3 mm away from the copper mold hot face. According to the process of the experiment, there are four stages (I–IV) in the whole temperature history. In stage I, the temperature increases rapidly from room temperature to 86 °C when mold flux A is used and to 78 °C when mold flux B is used. This is because the copper mold descends towards the molten bath, and it is heated up by the molten steel. In Stage II, to generate the initial solidified shell, the copper mold is kept in the molten bath for 5 s. So, the temperature reduces a little bit as the solidified shell increases the thermal resistance. The temperature increases again in stage III, and it results from the consecutive contact of fresh molten steel on the water-cooled mold wall at the meniscus during the casting stage. Finally, when the copper mold is withdrawn from the molten bath in Stage IV, the temperature starts to drop again gradually.

Generally, the temperature in the copper mold becomes lower (Figure 3a) when mold flux B is used, compared with that in Figure 3b when mold flux A is used. The highest temperature is 120 °C for mold flux B, while it is 160 °C for mold flux A. Moreover, the temperature fluctuation of these curves in Figure 3b becomes weaker than that in Figure 3a. For example, the amplitude of the temperature fluctuation in Stage III is about 2.8 °C (T5) for mold flux B, and it is 3.2 °C (T5) for mold flux A. All these phenomena are mainly due to the difference in thickness and structure of slag films when the two mold fluxes are used. The deeper analyses on the impact of thickness and structure of slag film on heat transfer from molten steel to mold wall will be stated in Section 3.2 and Section 3.3.

### 3.2. Slag Film Structure

**Figure 4:**
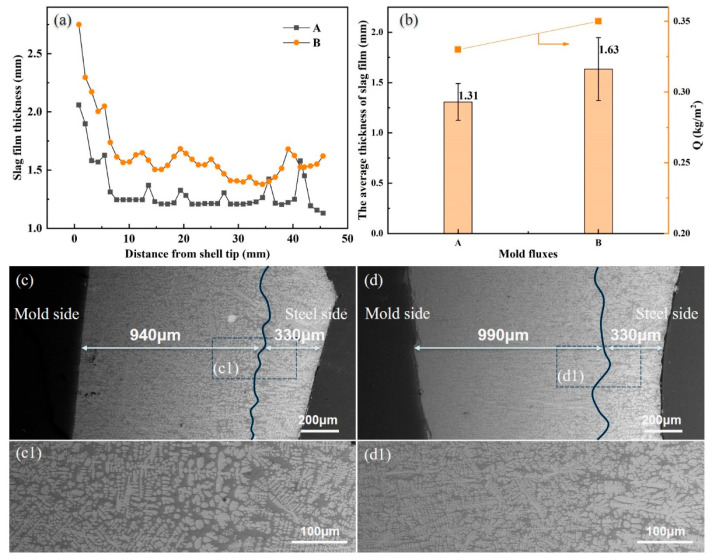
Slag films: (**a**) the measured thickness of the whole infiltrated slag film, (**b**) statistical slag film thickness and calculated slag consumption, (**c**) SEM images of slag film A, (**d**) SEM images of slag film B. (**c1**) Enlarged view of c, (**d1**) enlarged view of d.

As the heat transfer is affected by mold flux layers greatly, the slag film in the gap between the mold wall and solidified shell were stripped from the copper mold after the experiment. The thickness of the slag film from the meniscus to the bottom of the shell was measured using a vernier caliper, and its results are shown in Figure 4. The thickness of slag film at the meniscus is 2.1 mm and 2.75 mm for mold fluxes A and B, respectively, as shown in Figure 4a. However, it decreases rapidly at the distance away from the meniscus. The reason for that is because the slag film at the meniscus also belongs to part of the slag rim which is cooled in two dimensions from both the mold wall and air on the top of the mold flux.

The average thickness of the slag film and slag consumption of both mold fluxes were also calculated and are exhibited in Figure 4b. Equation (3) is the empirical formula for calculating the slag consumption in this simulation experiment.

(3) $$Q=0.55(\frac{60}{f}){\left(\eta V_{c}^{2}\right)}^{-0.5}+0.1$$

where, Q is the slag consumption, kg/m^2^; *f* is the mold frequency, cpm; *η* is the viscosity of mold flux, Pa·s; and $V_{c}$ is the casting speed, m/min.

From Figure 4, the average thickness of te slag film is 1.31 mm and 1.63 mm, and the consumption of the mold flux is 0.33 kg/m^2^ and 0.35 kg/m^2^, respectively. So, both the average thickness and consumption of mold flux B are larger than mold flux A. This is attributed to that both the melting point and viscosity of mold flux B are smaller than for mold flux A, which is beneficial for the infiltration of the mold flux into the gap between the mold wall and shell.

Figure 4c,d show the SEM images of slag films. It seems that crystals precipitate in whole slag films. But those near the mold side are of smaller size due to the rapid cooling from the mold wall, while the crystals near the solidified shell are larger, as they form in the liquid slag with a much slower cooling rate. The average thickness of the smaller size crystalline layer is 940 μm and 990 μm for mold flux A and B, respectively, but the thickness of the larger size crystalline layer is almost the same (330 μm). The thickness of the slag film and crystalline layer is affected by the viscosity, melting temperature and break temperature, and it also further affects the heat transfer across through the molten steel to the wall of the copper mold [19,20].

### 3.3. Heat Flux Analysis

**Figure 5:**
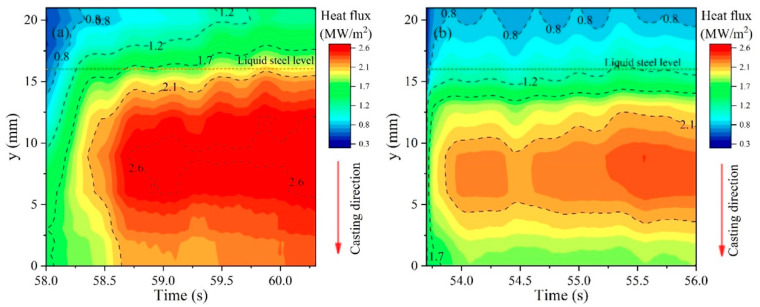
Heat flux through the hot surface of copper mold: (**a**) mold flux A, (**b**) mold flux B.

The heat flux contour map through the copper mold hot surface at the casting stage was calculated by 2DIHCP and is exhibited in Figure 5. The heat flux for mold flux B is generally smaller than that for mold flux A, as the color in Figure 5b is lighter than that in Figure 5a. The maximum heat flux for mold flux B is about 2.5 MW/m^2^, and that is about 2.7 MW/m^2^ for mold flux A. It is primarily attributed to the thicker slag film and thicker crystalline layer in slag film B, which results in an increase in the thermal resistance between the solidified shell and mold wall [21,22]. Moreover, in both cases, the maximum heat flux occurs in the region of approximately 5–10 mm below the meniscus, and this region is better for reflecting the early solidification phenomena associated with the mold oscillation and molten steel flow. Therefore, the heat flux at y = 9 mm or y = 8 mm is chosen as the typical heat flux for the subsequent characteristic analysis, providing a more comprehensive understanding of the early solidification phenomena related to the oscillation of the copper mold and solidification of molten steel.

**Figure 6:**
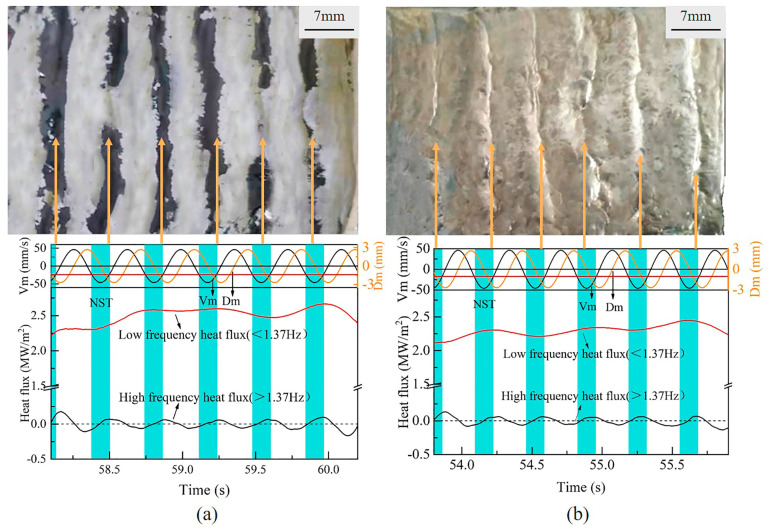
Relations among shell surface profile, low frequency heat flux and high frequency heat flux: (**a**) mold flux A, (**b**) mold flux B.

Figure 6 shows the corresponding relationship between the heat flux and profile of the solidified shell. The heat flux of low frequency and high frequency is decomposed from the original heat flux by an FFT filter. The threshold frequency of this filter is 1.37 Hz, which is equal to half of the mold oscillation frequency [23]. The heat flux with low frequency increases a little bit during the casting period, as the fresh molten steel consecutively touches and solidifies. The heat flux with high-frequency in both Figure 6a,b exhibits a strong correlation (one-to-one) with the oscillation mark on the shell surface. The cycle of the high-frequency heat flux is also kept the same as the mold oscillation. The main reason for those is because the high-frequency heat flux is from the oscillation of the copper mold, which is periodically in and out of the molten bath at the meniscus. In the meantime, the oscillation of the mold also causes the formation of an oscillation mark, through the overflowing or pushing back of molten steel to the tip of the shell. In addition, the amplitude of the fluctuation in the heat flux with both low frequency and high frequency for mold flux B is relatively smaller than that for mold flux A. This is also due to the thicker slag film and crystalline layer which has a stronger heat transfer control ability.

### 3.4. Solidified Shell Profile

**Figure 7:**
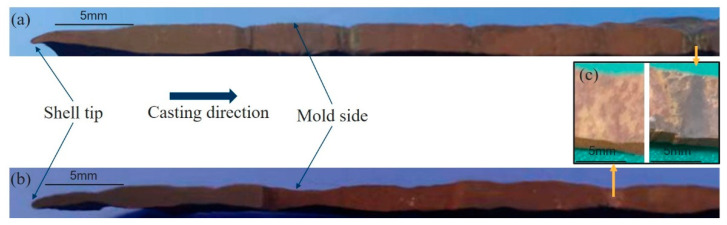
Profile of the solidified shell: (**a**) mold flux A, (**b**) mold flux B and (**c**) sampling.

The initial solidified shells were split along the casting direction for the profile analysis after the simulation experiment. Figure 7a,b show the cross-section of the shells for mold flux A and mold flux B, and Figure 7c is the cut-off parts from the shells for further analysis of entrapped slag.

**Figure 8:**
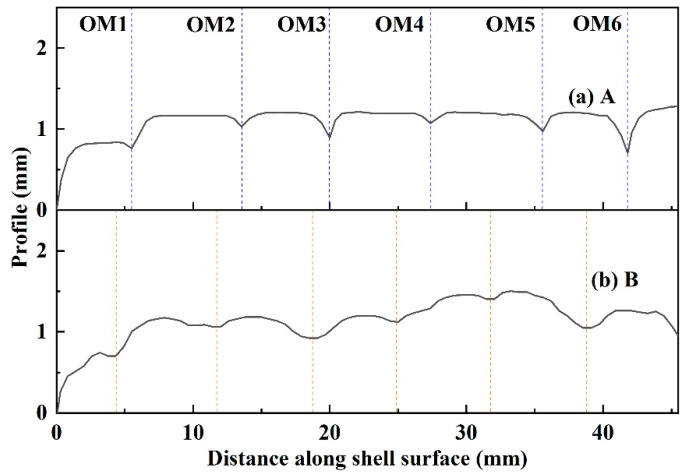
Measured profile of shell surface: (**a**) mold flux A; (**b**) mold flux B.

To observe the shape and distribution of the shell oscillation mark more clearly, the cross-section of shell in Figure 7a,b were characterized using the contact profilometer and shown in Figure 8. The difference is significant in the profile of the two shells. The shell for mold flux A exhibits sharper oscillation marks, while the shell for mold flux B has shallower oscillation marks. According to the previous research [24,25], there are two typical oscillation marks on the slab. One is the “hook” type, and the other is the “depression” type. From the profile of oscillation marks in Figure 8, it seems that the oscillation marks on mold flux A shell are more likely to be “hook” type, while those on mold flux A shell may be of “depression” type.

The depth of each oscillation mark was also measured and is listed in Table 4. The depth of oscillation marks on the shell for mold flux B (0.20 mm) is shallower than that for mold flux A (0.24 mm). As a deeper of oscillation mark indicates that the mold flux is easier to be captured, the oscillation mark when mold flux A is used is more severe than that for mold flux B. This means that a slag entrapment defect is more likely to be produced for mold flux A.

**Figure 9:**
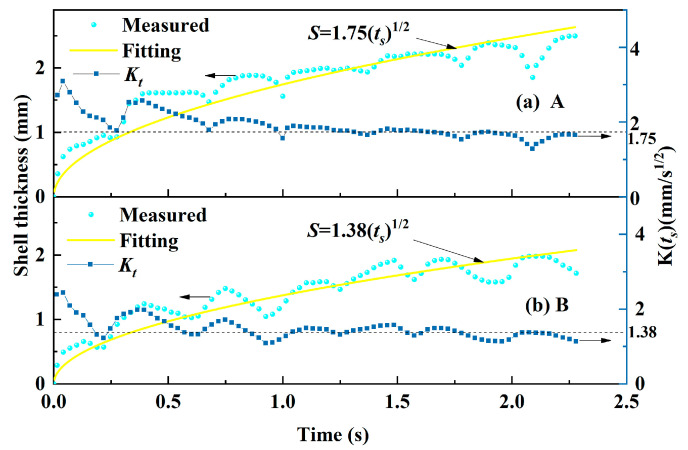
The change in shell thickness with time: (**a**) mold flux A and (**b**) mold flux B.

Figure 9 shows the change in shell thickness with time. In theory, the thickness (*S*) of the solidified shell depends on time *t_s_* for casting steel, and it is approximated by Equation (4) [26].

(4) $$S=K_{s}\sqrt{t_{s}}$$

where *t_s_* is the solidification time, s, and it is equal to *l*/*V_c_* (*l* is the distance from the tip of the shell, mm; *V_c_* is the casting speed, mm/s). *K_s_* is the solidification coefficient, mm/s^1/2^, and it is influenced by the copper mold cooling capacity, as well as molten steel superheat. So, the solidification coefficient *K_t_* can be calculated using Equation (5).

(5) $$K_{s}=S$$

The calculation result suggests that the solidification coefficient of the shell for mold flux B is 2.325 mm/s^1/2^, which is smaller than that for mold flux A (2.766 mm/s^1/2^). This also implies that the shell for mold flux B grows under a lower cooling-capacity condition due to the thicker slag film and crystalline layer which has a stronger heat transfer control ability. These results are consistent with the results of temperature and heat flux in the mold wall.

**Figure 10:**
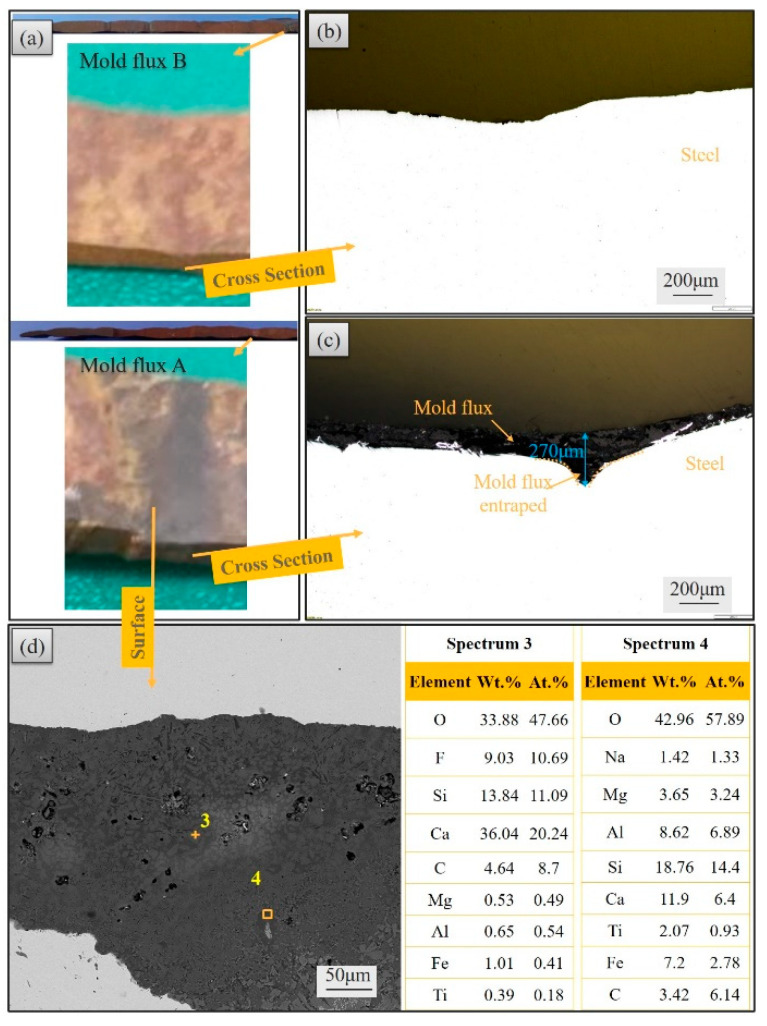
The entrapped slag at the location of oscillation mark: (**a**) sample, (**b**) metallographic images, (**c**) SEM image and (**d**) EDS results.

Samples in Figure 10a were taken from shells as shown in Figure 7 for slag entrapment analysis. Figure 10b,c are the cross-section of the oscillation mark location for mold flux B and mold flux A. It can be found that there are some substances, which are different from the steel substrate, appearing at the oscillation marks for mold flux A. However, the bareness steel substrate without entrapped substances occurs at the oscillation marks for mold flux B. The compositions of those substances at the oscillation marks for mold flux A were further identified by EDS. It shows that it is composed by Ca, Si, Al, Na and Mg (spectrum 3 and 4), which are the main components of the mold flux. In addition, the contents of C in spectra 3 and 4 are 4.63 and 3.43 wt.% due to the original C content in the mold powder and the C gathered in the slag rim at the meniscus.

Therefore, through the comprehensive analyses of the slag film in the gap, in-mold temperature, heat flux, oscillation mark on shell and entrapped slag at the location of oscillation mark, it can be concluded that the mechanism of the slag entrapment in the oscillation mark is mainly due to the formation of the slag film and crystalline layer with different thicknesses which affects the heat transfer (in-mold temperature and heat flux) from molten steel to the mold wall, and then leads to the production of a thicker or thinner solidified shell with a shallower or deeper oscillation mark. The deeper oscillation marks are more likely to be the “hook” type, which can easily capture the mold flux and produce an entrapped slag defect by overflow of molten steel at the meniscus.

## 4. Conclusions

The temperature in the mold, slag film structure and heat flux across the mold and solidified shell profile during the IF steel casting process were investigated using a high-temperature mold simulator. The following important conclusions are summarized:

(1) The temperature in the copper mold becomes lower when mold flux B was used, compared with that when mold flux A was used. Also, the temperature fluctuation becomes weaker for mold flux B than that for mold flux A.
(2) The average thickness of slag films for mold fluxes A and B are 1.31 mm and 1.63 mm. The slag consumption of them is 0.33 kg/m^2^ and 0.35 kg/m^2^, respectively. So, both the average thickness and consumption of mold flux B are larger than mold flux A.
(3) The heat flux for mold flux B is smaller compared with that for mold flux A, and the amplitude of fluctuation in heat fluxes with both low frequency and high frequency for mold flux B is relative smaller than that for mold flux A. It is due to the thicker slag film and crystalline layer which has a stronger heat transfer control ability.
(4) The shell for mold flux A exhibits sharper oscillation marks, while the shell for mold flux B has shallower oscillation marks. The sharper and deeper oscillation marks for mold flux A capture a substance composed of Ca, Si, Al, Na and Mg, which are the main components of the mold flux.
(5) From the results above, it can be concluded that the mechanism of slag entrapment in the oscillation mark mainly results from the formation of different thicknesses of the slag film and crystalline layer, which affects the heat transfer in the mold, and then leads to a deeper oscillation mark. Those deeper oscillation marks capture the mold flux and produce slag entrapment defect by overflow of molten steel at the meniscus.

## Figures and Tables

**Table 1:** Composition of the IF steel.

C	Mn	P	S	Al_tot_	Ti
≤0.0025	≤0.14	≤0.014	≤0.006	0.03–0.08	0.06–0.09

**Table 2:** Composition and properties of mold fluxes.

Mold Flux	CaO	SiO_2_	Al_2_O_3_	Na_2_O	MgO	F	Melting Temperature Range (°C)	Viscosity at 1300 °C (Pa·s)	Break Temperature (°C)
A	37–42	42–46	4–6	2.5–3.5	1–1.6	7	1113–1328	0.53	1171
B	32–39	36–43	4–6	3.5–6	2–3	4–7	1096–1323	0.44	1192

**Table 3:** Parameters of casting process.

Pouring Temperature (°C)	Casting Speed (m/min)	Frequency (cpm)	Stroke (mm)
1563	1.2	164 (2.73 Hz)	4.6

**Table 4:** The measured depth of oscillation marks.

		OM1	OM2	OM3	OM4	OM5	OM6	Ave.	STD
Depth (mm)	A	0.08	0.19	0.30	0.13	0.21	0.55	0.24	0.15
	B	0.08	0.20	0.40	0.07	0.15	0.30	0.20	0.12

## Data Availability

The data presented in this study are available on request from the corresponding author.
